# Seconeolitsine, the Novel Inhibitor of DNA Topoisomerase I, Protects against Invasive Pneumococcal Disease Caused by Fluoroquinolone-Resistant Strains

**DOI:** 10.3390/antibiotics10050573

**Published:** 2021-05-13

**Authors:** Jose Manuel Tirado-Vélez, David Carreño, David Sevillano, Luis Alou, José Yuste, Adela G. de la Campa

**Affiliations:** 1 Centro Nacional de Microbiología, Instituto de Salud Carlos III, Majadahonda, 28220 Madrid, Spain; jmtirvel@gmail.com (J.M.T.-V.); dcarreno@ic.ac.uk (D.C.); 2 Microbiology Division-Department of Medicine, Universidad Complutense de Madrid, 28040 Madrid, Spain; dsevill@med.ucm.es (D.S.); luisalou@med.ucm.es (L.A.); 3 CIBER de Enfermedades Respiratorias, 28029 Madrid, Spain; 4 Presidencia, Consejo Superior de Investigaciones Científicas, 28006 Madrid, Spain

**Keywords:** *Streptococcus pneumoniae*, DNA topoisomerase I inhibitor, seconeolitsine, resistance, invasive pneumococcal disease.

## Abstract

Antibiotic resistance in *Streptococcus pneumoniae* has increased worldwide, making fluoroquinolones an alternative therapeutic option. Fluoroquinolones inhibit the type II DNA topoisomerases (topoisomerase IV and gyrase). In this study we have evaluated the in vivo activity of seconeolitsine, an inhibitor of topoisomerase I. Levofloxacin (12.5 to 50 mg/kg) or seconeolitsine (5 to 40 mg/kg) were administered every 12 h during two days in mice infected with a serotype 8-resistant strain. At 48 h, a 70% protection was obtained with seconeolitsine (40 mg/kg; *p* < 0.001). However, survival with levofloxacin was 20%, regardless of the dose. In addition, seconeolitsine decreased bacteremia efficiently. Levofloxacin had higher levels in serum than seconeolitsine (Cmax of 14.7 vs. 1.6; *p* < 0.01) and higher values of area under the serum concentration-time curve (AUC_0-12h_ of 17.3 vs. 5; *p* < 0.01). However, seconeolitsine showed higher levels of time to peak concentration and elimination half-life. This is consistent with the higher binding of seconeolitsine to plasma proteins (40% and 80% when used at 1 µg/mL and 50 µg/mL, respectively) in comparison to levofloxacin (12% at 5 µg/mL and 33% at 50 µg/mL). Our results suggest that seconeolitsine would be a promising therapeutic alternative against pneumococcal isolates with high fluoroquinolone resistance levels.

## 1. Introduction

*Streptococcus pneumoniae* (the pneumococcus) is an important cause of morbidity and mortality worldwide, and it is a major etiological agent of community-acquired pneumonia, meningitis, and acute otitis. It is the leading cause of severe pneumonia across the developing world, causing one million deaths annually among children aged <5 years [1,2,3]. In spite of the development of pneumococcal conjugate vaccines and antimicrobial chemotherapy, the pneumococcus continues to be one of the major human pathogens, due in part to its high rate of resistance to some antibiotics and in part to the low coverage and partial inefficiency of available vaccines [1,2]. The large number of pneumococcal serotypes has made prevention of the disease through vaccination challenging, since vaccine introductions have been followed by changes in serotype prevalence. After the introduction of the pneumococcal conjugate vaccines, the incidence of invasive pneumococcal disease (IPD) declined drastically, coinciding with a decrease in penicillin resistance [4,5,6]. However, emergence of serotypes not included in the vaccines and associated with antibiotic resistance is of great concern worldwide [7,8,9]. 

When antibiotic treatment is considered, resistance to currently used drugs for the treatment of pneumococcal infections, including beta-lactams and macrolides, has spread worldwide [10]. DNA supercoiling is an appropriate antimicrobial target, given that it is involved in DNA replication, transcription, and recombination [11]. An adequate level of DNA supercoiling is maintained by the DNA topoisomerase enzymes. *S. pneumoniae* possess three of these enzymes: two of type II (gyrase and topoisomerase IV), and a single type I enzyme, topoisomerase I (Topo I). The respiratory fluoroquinolones (FQs), such as levofloxacin (LVX) and moxifloxacin (MXF), which target the type II topoisomerases, are used for the treatment of adult patients with community-acquired pneumonia [12]. Fluoroquinolone resistance in *S. pneumoniae* is maintained at low level (<3%) in Europe [13,14], although higher rates have been detected in Asia [15], as well as in Canada [16]. However, an increase in resistance in this bacterium would occur if FQ use were increased [17].

Identification of compounds specifically targeting the type I topoisomerase family is scarce. Cheng et al. [18] have described one phenanthrene alkaloid able to inhibit the relaxation activity of *Escherichia coli* Topo I. However, no significant inhibition in cell growth was observed. We have described novel alkaloid compounds (seconeolitsine and N-methyl-seconeolitsine) that inhibited *S. pneumoniae* Topo I activity in vitro at concentrations equivalent to those inhibiting cell growth (~10 µM). These drugs inhibited the growth of multidrug-resistant (including FQ-resistant) clinical isolates [19] without affecting human cell viability and are envisaged as new therapeutic candidates for the treatment of pneumococcal infections resistant to other antibiotics. Seconeolitsine (SCN), a phenantrene alkaloid that is semi-synthesized from boldine [19] inhibits both Topo I activity and growth of *S. pneumoniae* [19,20] and *Mycobacterium tuberculosis* [21]. In addition, SCN triggers a coordinated global transcriptional response in *S. pneumoniae* because of the increase of negative DNA supercoiling caused by inhibition of Topo I [20]. However, a definite proof of the in vivo activity of these drugs is still missing. In addition, the antimicrobial activity of these compounds has been recently demonstrated against multi-drug resistant isolates of *M. tuberculosis* and for the treatment of pneumococcal biofilms, confirming the therapeutic potential of these drugs against respiratory pathogens [21,22]. 

In this study, we investigated the efficacy of SCN in a mouse model of invasive disease in comparison with LVX. 

## 2. Materials and Methods

### 2.1. Bacterial Isolates, Growth, Typing, and Susceptibility Tests 

Isolates were serotyped by the Quellung reaction, a dot blot assay, and and/or by capsular sequence typing [23,24]. Genotyping was performed by pulse-field gel electrophoresis and multilocus sequence typing [25,26]. Five FQ-resistant isolates with characterized mutations were selected from a previous study [27]. Briefly, oligonucleotides parE398 and parC152 were used to amplify *parE* and *parC* QRDRs [27]. All isolates yielded fragments of 1.6 kb. These PCR fragments were sequenced as described [27]. Oligonucleotides gyrA44 and gyrA170 were used to amplify and sequence *gyrA* QRDRs [27]. 

Bacteria were grown in Todd-Hewitt broth supplemented with 0.5% yeast extract (Difco) to an optical density at 580 nm (OD580) of 0.4 to 0.5 corresponding to ca. 10^8^ CFU/mL. Single-use aliquots were stored at −80 °C in 10% glycerol to use in mice infections as previously described [28,29,30,31,32]. Antimicrobial susceptibility was tested by broth microdilution, according to the Clinical and Laboratory Standards Institute guidelines. *S. pneumoniae* R6 was included as a quality control. Powders of known potency of LVX and MXF were purchased from Sigma. SCN was synthesized as previously described [19]. Briefly, for MIC determination, pneumococcal strains were grown in a casein hydrolysate-based medium with 0.3% sucrose as an energy source (AGCH medium). A 96-well plate was used including broth containing different concentrations of antibiotics. Plates were inoculated with a standardized suspension of the pneumococcal strains followed by incubation at 37 °C with 5% CO_2_ for 18–24 h. The minimal inhibitory concentrations (MICs) were determined by observing the lowest concentration of the agent that inhibited visible growth of the bacterium. 

### 2.2. Animal Model Experiments 

BALB/c female mice (8–12 weeks old) weighing 20 g were purchased from Harlan Laboratories (Barcelona, Spain). These mice have been shown to be a good animal model to explore the protective activity of several antibiotics, including FQs [28,31,32]. The lethal dose of bacteria producing a 100% mortality rate (LD_100_) over a period of 7 days was investigated by inoculating different concentrations of bacteria diluted in phosphate-buffered saline (PBS) pH 7.3, by the intraperitoneal route as previously described [28,31,33]. To evaluate the protection level of SCN and LVX, animals were challenged with the LD_100_ 1 h before antibiotic treatment [28,31,33,34,35]. Different concentrations of antibiotic (10, 20, and 40 mg/kg for SCN; 12.5, 25, and 50 mg/kg for LVX) were administered two times a day over 48 h by the subcutaneous route. The doses, interval, and schedule chosen were based in previous studies investigating the protective activity of FQs against pneumococcal sepsis in mice [34,36,37]. Seconeolitsine stock solution was dissolved in dimethyl sulfoxide (DMSO) and the doses tested for protection in animals were prepared in PBS containing 1% DMSO. Animals in the lethal control groups were infected with *S. pneumoniae* and received the antibiotic solvent (PBS−1% DMSO) instead of SCN or LVX. The toxicity control for each antibiotic (no infection group) only received SCN or LVX. Animal experiments were performed in groups of at least 5 mice and were repeated twice.

### 2.3. Determination of Bacteria in Blood 

Bacteremic profiles (colony counts in blood) were determined from mice treated with SCN and LVX, at 24 h and 48 h post-infection, when the majority of mice were alive. To collect blood samples, volumes of 6 µl were obtained from the tail vein, as previously described [28,29,30,31,33,38], resuspended in PBS and plated onto blood agar plates at 37 °C in 5% CO_2_ for colony counts determination.

### 2.4. Determination Antibiotic Concentrations in Serum 

Groups of two mice for each antibiotic and time-point were treated with a single subcutaneous dose of 40 mg/kg of SCN or 50 mg/kg of LVX. Blood samples were collected at 15 and 30 min and 1, 2, 4, 6, 8, 10, 12, 14, 18, and 24 h, centrifuged for 10 minutes at 2000 rpm, and the sera from the different mice were stored at −80 °C until drug assay determination. 

Seconeolitsine concentrations were measured by high-pressure liquid chromatography analysis (HPLC). Chromatographic separation was performed using SunFire C18 column (150 × 4.6 mm; Waters Corporation, MA, USA) with trifluoroacetic acid and acetonitrile in the ratio 70:30 (*vol.*/*vol.*) as the mobile phase. The solvent flow rate was 1.0 mL/min with a runtime of 20 minutes. The mobile phase was filtered through 0.45 µm prior to use. Standards ranging from 0−1.000 µg/mL were prepared in HPLC grade methanol. Sample dilutions, prepared in mouse serum, were injected into the column at a constant volume of 20 µl and were tested in duplicate. The detection wavelength was 270 by UV absorbance using a Waters UV–Visible 2489 module (Waters Corporation). The column was maintained at room temperature.

Levofloxacin concentrations were determined by bioassay using *E. coli* NCTC 10418 as indicator organism, as previously described [39]. Plates containing a lawn of microorganism were incubated for 18–24 h at 37 °C. Standards and dilutions were prepared in mouse serum in a range from 0.03–8 µg/ mL.

### 2.5. Protein Binding 

Binding to serum proteins was determined by the ultrafiltration method (36) using the Centrifree micropartition system (Amicon Bioseparations, Millipore, Tullagreen, Ireland) with concentrations in murine plasma of 1 and 50 μg/mL for SCN and 5 and 50 μg/mL for LVX. Pre-filtered samples and ultrafiltrates recovered were measured by HPLC or bioassay, as described above. Percentage of SCN or LVX bound to proteins in mouse serum was calculated using the expression: [antibiotic in pre−filtered samples] − [antibiotic in ultrafiltrated samples]/[antibiotic in pre-filtered samples] × 100 as previously described [40].

### 2.6. Drug Pharmacokinetics and Pharmacokinetic/Pharmacodynamics (PK/PD) Parameters

Pharmacokinetic (PK) parameters measured included peak level (C_max_), time peak level (T_max_), area under the concentration time-curve from 0 to last (C_last_) measured concentration (AUC_last_), and elimination half-life (t_1/2_). These parameters were estimated for both the total concentrations and the free fraction concentrations and were determined by using protein binding values, by a non-compartmental approach with the Phoenix WinNonlin program (version 6.2, Certara, NJ, USA). Pharmacodynamic (PD) index values (AUC/MIC ratio and T>MIC) were calculated over a period of 24 h. T>MIC for total and unbound concentrations (*f*T>MIC) were determined by a non-compartmental approach for PD data (Phoenix WinNonlin, model 220). AUC/MIC and *f*AUC/MIC were calculated as twice the AUC_0–last_ (or *f*AUC_0-last_), since C_last_ was detected at times less than 12 h. 

### 2.7. Statistical Analysis 

Statistical analysis was performed by using two-tailed Student’s *t*-test (for two groups), whereas analysis of variance (ANOVA) was chosen for multiple comparisons. Survival was analyzed by the log-rank test. GraphPad InStat version 5.0 (GraphPad Software, CA, USA) was used for statistical analysis. Differences were considered statistically significant with *p* < 0.05 (*), highly significant with *p* < 0.01 (**) and extremely significant with *p* < 0.001 (***).

### 2.8. Ethics Statement

Animal experiments were performed at ISCIII in accordance with Spanish legislation (RD 1201/2005, RD 53/2013) and EU regulations (218/63/EU). All animal experiments were approved by the Animal Care and Use Committee of ISCIII (CBA PA 52_2011-v2 and PROEX 218/15).

## 3. Results

### 3.1. Virulence among FQ-Resistant Clinical Isolates Varies within Serotype

To investigate the LD100, we selected five FQ-resistant clinical isolates of four different serotypes from among those detected in a previous study, including Spanish isolates from year 2006 [27]. These strains were resistant to all FQs tested and also had resistance associated with other antibiotics (Table 1). All isolates were of serotypes (8, 15A, 16F, and 33F) not included neither in the 10-valent or 13-valent pneumococcal conjugate vaccines. Three of them (CipR45, CipR57, and CipR72) belong to the Sweden15A-ST63 genotype, a major genotype among FQ-resistant isolates of the post-PCV7 vaccine isolates. In terms of virulence, isolates CipR45 and CipR57 of serotype 8 were able to produce 100% mortality within the first 48 hours, with LD100 of about 101 CFU/mouse (Table 1), whereas strains of the other three serotypes were not virulent at the highest dose evaluated of 5 × 10^6^ CFU/ mouse (Table 1). Based on these results, isolates CipR45 (low-level FQ-resistant, carrying a ParC with a S79F change) and CipR57 (high-level FQ-resistant, carrying ParCS79F and GyrAS81F changes) were selected for further experiments. The MICs of SCN for these strains were similar (5.2 µg/mL for CipR45 and 2.6 µg/mL for CipR57; Table 1).

### 3.2. In Vivo Efficacy Studies

**Figure 1:**
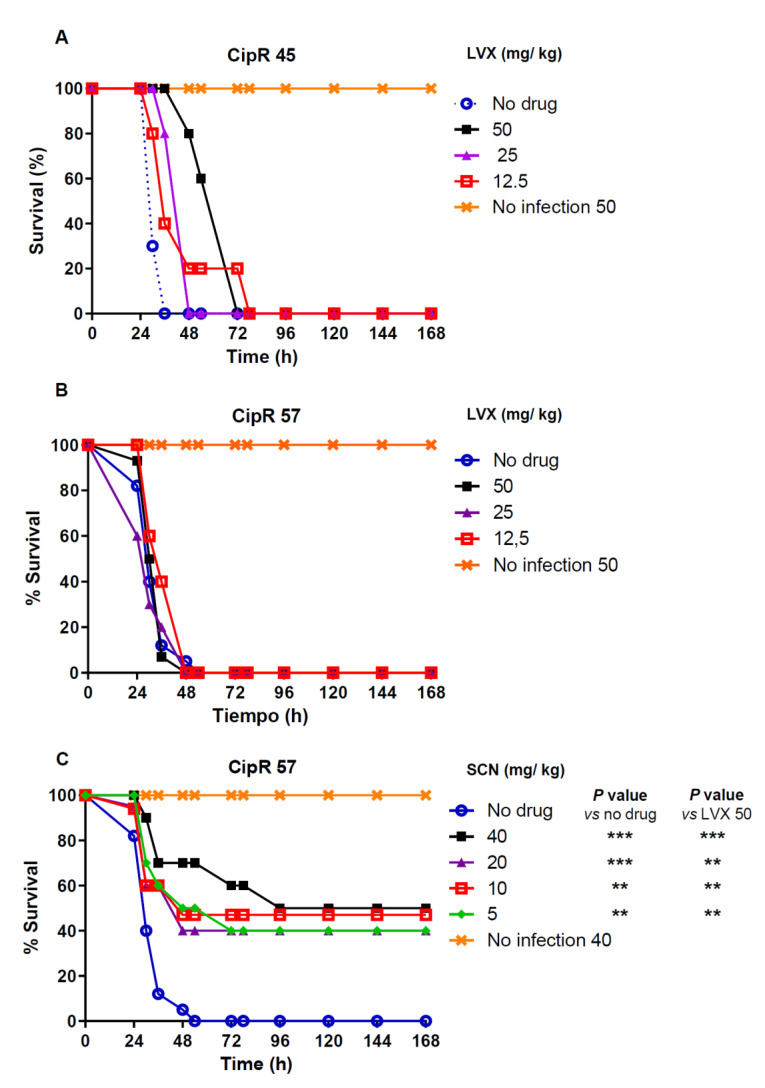
Percentage survival of groups of 5 mice intraperitonealy infected with two times the MLD (1 × 10^2^ CFU/mouse) of isolate CipR45 (**A**) or CipR57 (**B**,**C**). Experiments were followed over a 7-day period. Antibiotic treatment or placebo was administered subcutaneously every 12 h during the first 48 h. The doses of each antibiotic administered are indicated for LVX (A and B) or SCN (C). Data are the average of 2 (for SCN 40 mg/kg) to 4 independent replicates. The long rank test was used for survival curve comparison between the no drug group and the groups treated with the indicated SCN concentrations. *p* values are: ** *p* < 0.01; *** *p*< 0.001.

Protection mediated by LVX was tested in a low-level resistant isolate (CipR45, LVX MIC of 2 µg/mL) and in a high-level resistant isolate (CipR57, LVX MIC of 16 µg/mL). Treatment with LVX concentrations ranging from 12.5 to 50 mg/kg produced a delay in the death course of the mice in a dose-dependent manner when the infection was caused by the CipR45 isolate (Figure 1A). At 36 h post-infection, a survival of 100%, 80%, and 40% was observed in the groups treated with LVX concentrations of 50 mg/kg, 25 mg/kg and 12.5 mg/kg, respectively. At 78 h post-treatment all mice succumbed to the infection (Figure 1A). However, when the high-level resistant CipR57 was used, almost no delay in the death dynamics was observed at any of the concentrations investigated and all the animals died within the first 48 h post-infection, regardless of LVX exposure (Figure 1B). 

To explore the putative protection of new compound SCN, the efficacy of this drug was tested in mice treated with 5, 10, 20, and 40 mg/kg of SCN after infection with the high-level resistant CipR57 strain. At 36 h post-infection, survival values of 70% (40 mg/kg), and 60% (20 mg/kg to 5 mg/kg) were observed (Figure 1C). After 7 days, survival rate among the different groups treated with SCN was still between 40% and 50%. These results indicate that SCN not only was able to induce a significant delay in terms of mortality but was even able to allow the long-term survival of half the animals against pneumococcal sepsis.

**Figure 2:**
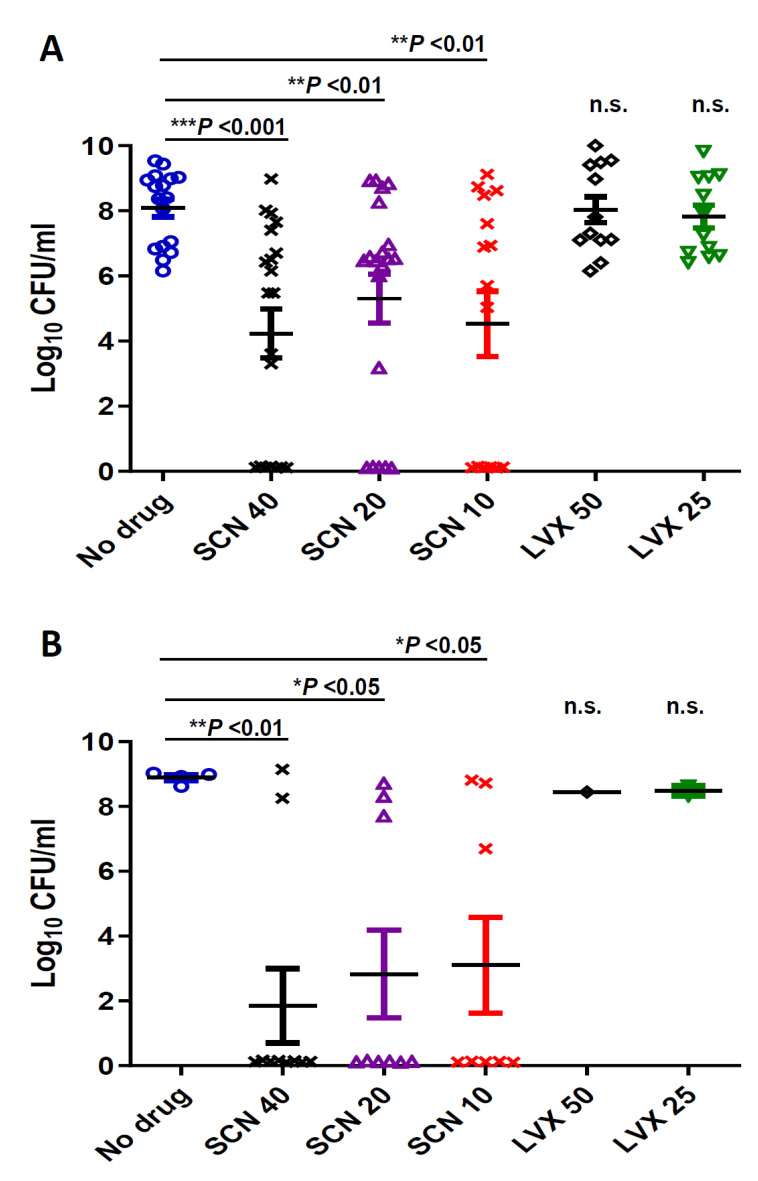
Determination of bacterial counts in the blood of at least 10 mice. To measure the level of bacteria in the bloodstream, blood samples were collected from the tail of the mice of each group at (**A**) 24 h or (**B**) 48 h after infection, plated on blood agar and incubated at 37 °C in 5% CO_2_ for 24 h. Data are the average of 3 independent replicates. The *t*-test was used to estimate the difference between the treated groups and the untreated (no drug) group. The symbols for *p* values are: * *p* < 0.05; ** *p* < 0.01, *** *p* < 0.001 and n.s.: not significant. For multiple comparisons of the different doses of SCN (one-way ANOVA with a post hoc Dunnett test); *p* < 0.01 and *p* < 0.05 for 24 h (**A**) and 48h (**B**) respectively. The comparison of different doses of SCN with LVX was statistically significant at 24 h when the majority of mice were alive ( *p*< 0.01 for SCN 40 vs. LVX 50 and 25; *p* < 0.05 for SCN 20 vs. LVX 50 and 25 and *p* < 0.01 for SCN 10 vs. LVX 50 and 25).

Seconeolitsine caused a decrease of bacterial counts in blood. Bacterial loads at 24 and 48 h post-infection were determined for the experiment in which the high-level LVX resistant CipR57 strain was used. Treatment with doses of 25 mg/kg and 50 mg/kg of Levofloxacin did not reduce bacterial levels in blood compared to the untreated group at any of the time-points investigated (Figure 2). However, treatment with different doses of SCN significantly reduced bacterial load (Figure 2). In this sense, whereas untreated animals or mice treated with LVX had around 10^8^ CFU/ mL bacterial levels in blood at 24 h, mice treated with SCN carried lower quantities of bacteria, with levels around 10^4^−10^5^ CFU/mL (Figure 2A). This effect was even more dramatic at 48 h post-infection, indicating that SCN has an important effect in preventing bacterial replication within the blood (Figure 2B). High levels of bacterial counts were found in blood of mice treated with LVX, whereas a significant proportion of mice treated with SCN were free of bacteria within the first 48 h, demonstrating that SCN showed bactericidal activity in vivo (Figure 2). Overall, our results confirm that SCN can induce bacterial clearance from the systemic circulation against sepsis caused by pneumococcal strains harboring high levels of resistance to FQs. 

### 3.3. Pharmacokinetic and PK/PD Parameters

Different flow rates, solvent systems, and ratios for the mobile phase were studied in order to obtain a sharp SCN peak with a shorter retention time. The solvent system comprising trifluoroacetic acid and acetonitrile 70:30 vol./vol. at a flow rate of 1 mL/min gave optimal separation at a retention time of approximately 7 to 8 min. The assay was linear over the range 0–200 µg/mL (r2 = 0.9987), with a lower detection limit of 0.25 µg/mL. Interday coefficient of variation (CV) was 4.5%. The bioassay for LVX was linear over the range tested (r2 = 0.995) with a lower limit of detection of 0.03 µg/mL. Interday and intraday CVs were lower than 5.2%.

**Figure 3:**
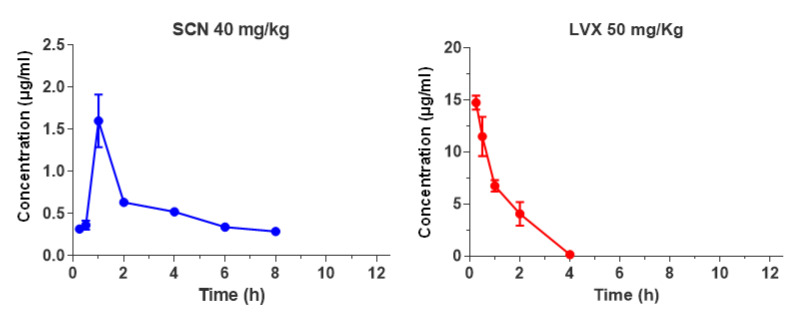
Serum drug concentrations (µg/mL) of SCN and LVX after single dose administration of 40 mg/kg or 50 mg/kg, respectively.

The mean serum concentration-time profiles of single subcutaneous doses of 40 mg/kg of SCN or 50 mg/kg of LVX (the highest doses used in efficacy studies) in non-infected mice are shown in Figure 3. 

Pharmacokinetic and PK/PD parameters estimated for both antibiotics are listed in Table 2. The half-life of SCN was prolonged, 7.8 h, in comparison with the half-life of LVX, 0.7 h, but the peak dose values for SCN were significantly lower than those observed for LVX (1.6 vs. 14.7 µg/mL, respectively). The protein binding level was concentration dependent for both antimicrobials, ranging from 40% to 80% for SCN at concentrations of 1 and 50 µg/mL, and from 12% to 33% for LVX at concentrations of 5 and 50 µg/mL, respectively. A protein binding value of 40% for SCN and 12% for LVX was chosen for determination of PK/PD parameters for free drug, since these percentages were more closely related to drug levels achieved by SCN or LVX at the doses tested. As shown in Table 2, magnitudes of PK/PD parameters of SCN exposed in our murine model against CipR45 and CipR57 were very low (*f*AUC/MIC ≤ 1.9 h and *f*T>MIC = 0). This is consistent with the free serum SCN levels maintained below the MIC achieved during the entire dosage interval for both CipR isolates. In contrast, LVX *f*AUC/MIC ratios derived from the dose tested were very low (≤15.2 h) even when the low-level FQ-resistant isolate was considered (Table 2).

## 4. Discussion

To evaluate the in vivo activity of SCN, we tested several clinical isolates with well-characterized mechanisms of FQ resistance. The most virulent isolates were two isolates of serotype 8 belonging to the Sweden15A-ST63 genotype. Serotype 8, which is not included either in PCV7 or PCV-13 vaccines, has been responsible for some invasive outbreaks reported in Spain in recent years, both in healthy individuals [41] and HIV patients [42]. These isolates were originated by recombination of the loci encoding capsule 8 into the multi-drug resistant Sweden15A-ST63 clone [26]. The inclusion of clinical isolates of serotype 8 to test the antimicrobial effect of SCN is relevant from the epidemiological perspective, because serotype 8 is currently the most frequent cause of pneumococcal disease in the adult population and is rising in children [7,8,9,43,44].

We observed that treatment with LVX exerted some protection against infection by CipR45, in accordance with its low level of resistance to LVX. This is consistent with previous studies showing that different FQs induce certain degrees of protection against pneumococcal strains with resistance levels to LVX ≤ 2 µg/mL [34,36]. However, the LVX exposures against the CipR45 isolate used in our animal model were insufficient for efficacy, as confirmed by the low rates of survival beyond 48 h of treatment. Data from non-neutropenic mice infected with *S. pneumoniae* estimated that LVX fAUC/MIC ratios greater than 25 h are associated with a 90% survival; i.e., efficacy when mortality is used as efficacy endpoint [45,46]. Higher *f*AUC/MIC ratios (>100 h) would be required to prevent the emergence of LVX-resistant mutants in environments with first step bacterial mutants [47]. These magnitudes are substantially greater than the LVX exposures used in this study (*f*AUC/MIC ≤ 15.2 h; *p* < 0.01), even when the highest LVX dose was tested. As expected, when isolate CipR57 containing a high level of resistance to LVX was used, no protection was observed. These results confirmed previous findings by other authors demonstrating poor efficacy of treatment with LVX against sepsis caused by *S. pneumoniae* strains with *parC* and *gyrA* mutations and also support the validity of the animal model used [34,36]. 

As an alternative antibiotic strategy to fight pneumococcal sepsis caused by clinical isolates with high levels of resistance to FQ, we used SCN as a novel drug targeting a new target, Topo I. In this sense, we have demonstrated that the antimicrobial activity of this compound was very high against different multidrug pneumococcal resistant strains including those with high levels of MIC to FQs [19]. In addition, SCN is effective against multidrug resistant strains of *M. tuberculosis* and reduces the adherence and thickness of pneumococcal biofilms [21,22]. In this study, SCN showed a high capacity of protection against *S. pneumoniae* infection, even at the lower dose tested, 5 mg/kg (*p* < 0.01). After 7 days, survival rate was still of 40% to 50%, regardless of the dose of SCN administered (*p* < 0.01). This was contrary to what happened in LVX-treated mice, which all died within the first 48 h. The survival was directly related to the ability of SCN to reduce the number of viable pneumococci present in the plasma of the mice, both at 24 and 48 h after infection. This number was significantly lower in those mice treated with 40 mg/kg of SCN compared with a control group (*p* < 0.001) and also with the mice group treated with LVX (*p* < 0.001). These results confirm previous studies by our group, demonstrating that SCN has antibacterial activity both in vitro and in vivo and might be a promising alternative against *S. pneumoniae* strains with high levels of resistance to FQs [19]. 

The pharmacokinetic study of SCN demonstrated a long half-life in mice and peak concentrations below the MIC of strains CipR45 and CipR57 after the administration of a 40 mg/kg single dose. From a pharmacodynamic perspective, these observations are of great interest, since very low SCN exposures (*f*AUC/MIC ratios ≤ 2.3 h) were associated with high survival rates of mice after 48 h of treatment (*p* < 0.01).

In addition, SCN at the largest concentration used (40 mg/ kg) was not harmful as all non-infected mice were alive and their aspect remained healthy after 7 days (Figure 1C). Breathing, hair, eyes, appetite, movement, and weight were all normal parameters during the 7 days of follow-up. This is consistent with the very low cytotoxic activity of different alkaloids targeting Topo I that exhibit efficient antitrypanosomal activity [48,49]. Hence, since SCN response increased in a dose-dependent manner, a higher dose could improve the effectiveness of SCN when tolerable. Although SCN-derived metabolites were not detected in murine plasma by HPLC, their presence cannot be discounted since the quantification of SCN could not be performed by microbiological methods, probably due to poor hydrosolubility of this molecule (data not shown). Thus, the pharmacological activity of SCN in this model cannot be attributed solely to the parent compound.

Overall, we have proved that SCN is effective in a mouse model of pneumococcal bacteremia. Dose-response studies using higher tolerable SCN doses are guaranteed to provide the magnitude of PK/PD parameters required for efficacy on pneumococcal isolates. Topo I has an essential role in supporting transcription in *S. pneumonia*e. We have shown that Topo I and RNA polymerase physically interact in vitro and colocalize at gene promoters in vivo. Binding of each of these enzymes to promoters was prevented by the specific inhibition of the other enzyme, either by rifampicin (RNA polymerase) or by SCN (Topo I), supporting a role for Topo I in RNA polymerase transcription [50]. These results allowed envisaging SCN as a new drug for the treatment of diseases caused by infection with multi-drug resistant strains of *S. pneumoniae*.

## 5. Conclusions

Antibiotic resistance and reducing its impact on public health is one of the top priorities worldwide in the fight against infectious diseases. Hence, we demonstrate that seconeolitsine, which is an inhibitor of Topo I, is effective against systemic infection of *S. pneumoniae*. Administration of this drug was able to control bacterial replication within the systemic circulation, leading to increased survival rates. This study has analyzed the antimicrobial activity of this compound against pneumococcal sepsis, suggesting the potential of seconeolitsine as a promising therapeutic agent against pneumococcal infection. 

## Figures and Tables

**Table 1:** Phenotypic and genotypic properties of the isolates used in this study.

Isolate	Type ^a^	MIC (µg/ mL) ^b^:				Pattern ^c^	QRDR Statuses ^d^:			MLD ^e^
		SCN	CIP	LVX	MXF		ParC	ParE	GyrA	
CipR5	33F	5.2	16	8	2.00	EClCip	D83N	None	E85K	Avirulent
CipR15	16F	2.6	32	16	4.00	SxTCip	S79F	None	S81F	Avirulent
CipR45	8	5.2	4	2	0.25	TEClCip	S79F	None	None	≥5 × 10^1^
CipR57	8	2.6	64	16	4.00	TECip	S79F	None	S81F	≥5 × 10^1^
CipR72	15A	5.2	64	32	4.00	PTEClCip	S79F	E474K	S81F	Avirulent

^a ^Serotype; ^b^ SCN, seconeolitsine; CIP, ciprofloxacin; LVX, levofloxacin; MXF, moxifloxacin; ^c^ Pattern of resistance: P, resistant to penicillin (MICs of 0.12 to 4 µg/ mL); T, resistant to tetracycline (MICs ≥ 4 µg/ mL); C, resistant to chloramphenicol (MICs ≥ 8 µg/ mL); E, resistant to erythromycin (MICs ≥ 0.5 µg/ mL); Cip: resistant to ciprofloxacin (MICs ≥ 4 µg/ mL). ^d^ QRDR, Quinolone-resistance determining regions. Only amino acid changes involved in fluoroquinolone resistance are indicated. ^e^ MLD, minimal lethal dose that produced a 100% mortality rate at day 2.

**Table 2:** Pharmacokinetic (mean ± standard deviation) and PK/PD parameters of seconeolitsine and levofloxacin measured in pooled sera obtained after subcutaneous inoculation of a single dose of 40 mg/kg or 50 mg/kg respectively.

PK Parameters	Seconeolitsine	Levofloxacin	*p* Values
C_max_ (µg/ mL)	1.6 ± 0.3	14.7 ± 0.7	<0.01
C_min_(µg/ mL)	0.3 ± 0.04	0.1 ± 0.0	<0.05
T_max_ (h)	1.0 ± 0.0	0.25 ± 0.0	<0.001
t_1/2_ (h)	7.8 ± 2.4	0.7 ± 0.0	0.054
T_last_ (h)	9.0 ± 1.4	4.0 ± 0.0	<0.5
AUC_0-12h_ (µg/mL×h)	5.0 ± 0.0	17.3 ± 1.5	<0.01
AUC_0-24h_ (µg/mL×h)	10.0 ± 0.1	34.6 ± 2.9	<0.01
PK/PD parameters			
Strain CipR45; MIC (µg/ mL)	5.2	2	
*f*AUC/MIC (h)	1.61 ± 0.0	15.2 ± 1.3	<0.01
*f*T>MIC (%)	0.0	24.2 ± 2.8	-
Strain CipR57; MIC (µg/ mL)	2.6	16	
*f*AUC/MIC (h)	2.3 ± 0.0	1.9 ± 0.16	0.074
*f*T>MIC (%)	0.0	0.0	-

## Data Availability

The data presented in this study are contained within the article.
